# Large inguinal synovial sarcoma mimics a vascular lesion: A case report and literature review

**DOI:** 10.1016/j.ijscr.2020.10.050

**Published:** 2020-10-22

**Authors:** Wei-Chieh Chen, Po-Chien Wu, Chun-Yu Lin, Ting-En Tai

**Affiliations:** a Department of Urology, Taipei Medical University Hospital, No. 252, Wuxing Street, Taipei, 110, Taiwan; b Department of Medical Education, Taipei Veterans General Hospital, 112, Taiwan; c Department of Medical Imaging, Taipei Medical University Hospital, No. 252, Wuxing Street, Taipei, 110, Taiwan

**Keywords:** SS, synovial sarcoma, CT, computed tomography, STS, soft tissue sarcomas, Fish, fluorescence in situ hybridization, Synovial sarcoma, Soft tissue sarcoma, Inguinal tumor, Case report

## Abstract

**Introduction:**

Synovial sarcoma (SS) is one of soft tissue sarcomas (STS), characterized by t(X;18)(p11;q11) chromosomal translocation. Clinical diagnosis of SS in groin is difficult owing to rarity and various manifestations. We reported a rare and interesting case of inguinal biphasic SS with initial impression of a vascular lesion.

**Presentation of case:**

We reported a 72-year-old man who presented with a right inguinal progressively enlarged mass for 5 years. The mass became ulcerative with active bleeding after blunt trauma. Computed tomography (CT) showed a large heterogeneous tumor with focal vivid enhanced area, and an inguinal vascular lesion was impressed. Angiography revealed faint tumor stain without contrast extravasation or aneurysm lesion. Surgical resection of the tumor was performed. Final pathology and fluorescence in situ examination (FISH) demonstrated biphasic SS with SS18 gene rearrangement. No evidence of recurrence after one-year follow-up.

**Discussion:**

SS constitutes 5%–10% of STS. The most primary sites are extremities (68.7%) and trunk (15.7%). Treatment options for SS include surgery, chemotherapy, radiotherapy, targeted therapy, and novel therapies. In our case, the initial impressions of large inguinal tumor were hematoma or pseudoaneurysm. Preoperative angiography assisted in differentiating the lesion, evaluating tumor vessels, and preventing bleeding by embolization. Our patient recovered satisfactorily after surgical excision without major complications.

**Conclusion:**

Primary inguinal SS is an extremely rare tumor which can present as a vascular lesion. Diagnosis is confirmed by pathology and FISH exam. Further studies are required to determine the manifestation and management of SS.

## Highlights

• Synovial sarcomas mostly affect adolescents and young adults with slight male predominance.
• The cellular origin of SS is disordered mesenchymal stem cells that have undergone self-renewal dysregulation mediated by the SS18-SSX fusion protein.
• Novel therapies targeting molecular and genetic pathways of SS are under research, including SYT-SSX-derived peptide vaccine, retinoic acid and its derivatives.
• Various manifestations and rarity of SS could mimic vascular lesion which mislead the diagnosis.

## 1 Introduction

Synovial sarcoma (SS) is a high-grade soft tissue sarcoma (STS) predominantly found near large joints in the extremities. SS can occur at any age, but it commonly occurs in teenagers and young adults aged 15–40 years [1]. The t(X;18)(p11;q11) chromosomal translocation results in SS18 gene rearrangement and the formation of the SYT-SSX fusion protein in SS [2]. From a histological perspective, SS is monophasic or biphasic, which presents a spindle-cell component with a variable degree of epithelial differentiation. Factors such as tumor size, histological subtype, metastases, and lymph node involvement affect the prognosis of patients with SS.

Numerous unusual locations of SS have been reported and confirmed by using molecular and immunohistochemistry techniques Cases of SS primarily originating in inguinal areas are rare in English language literature [3]. Many lesions can attribute to groin tumor. The initial signs and symptoms of SS is hard to distinguish from other lesions. There is no standard treatment for inguinal SS. Herein, we reported a rare case of large inguinal SS and we reviewed updated information on multimodal therapy of STS.

## 2 Presentation of case

**Fig. 1:**
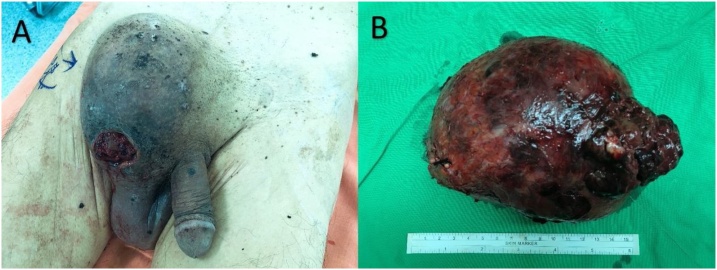
(A) Right inguinal large mass with ulcerative wound and bleeding. (B) Gross appearance of the tumor after surgical resection.

**Fig. 2:**
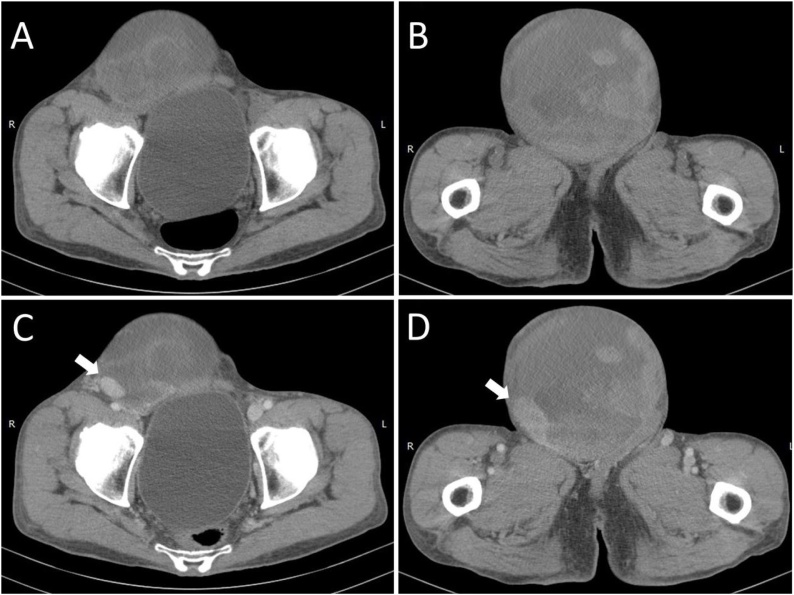
(A, B) Non-contrast CT image revealed a large right inguinal tumor with heterogeneous density (C, D) Contrast-enhanced CT image displayed vascular like lesions inside the tumor, raising the suspicion of pseudoaneurysm or active bleeding (white arrow).

**Fig. 3:**
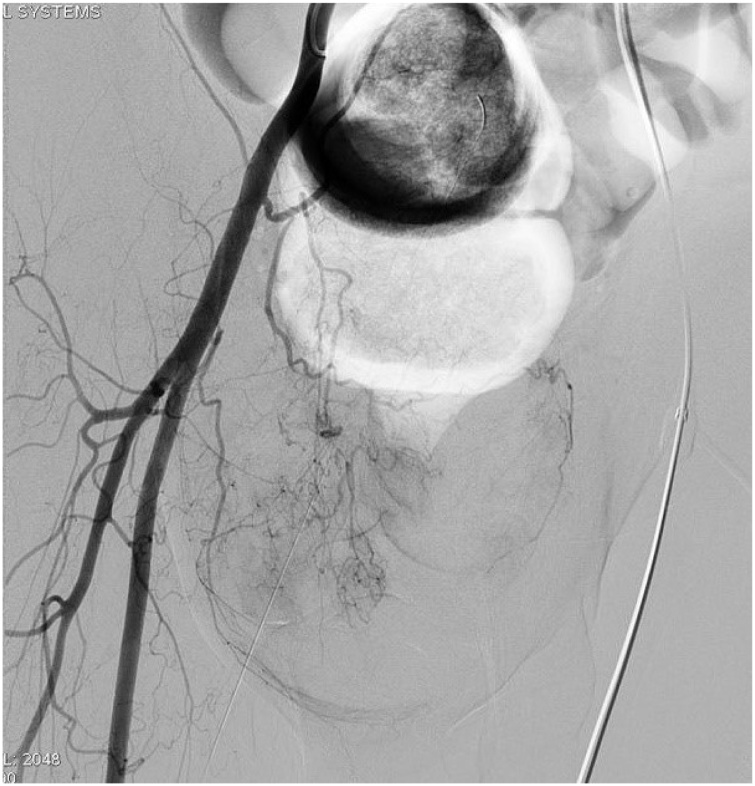
Pelvic angiography image depicted a hypervascular tumor with abundant blood supply.

A 72-year-old man presented with a right inguinal progressively enlarged mass existing for 5 years. The tumor surface became ulcerative and painful with active bleeding for 2 weeks after he hit against a desk at right groin tumor (Fig. 1). Physical examination revealed a 15cm × 15cm large hard mass with ulcerative wound and tenderness. His medical history was unremarkable. Laboratory examination showed normocytic anemia (red blood cell count: 2.59 × 10^6^ uL; hemoglobin level: 8.9 g/dL; and hematocrit level: 26.2%). Ultrasound examination revealed a heterogeneous mass, diameter up to 15 cm, with abundant blood supply, as assessed by Doppler mode. Computed tomography (CT) revealed a well-circumscribed right inguinal heterogeneous tumor, measuring up to 15 cm, with a vivid enhanced area, implying the possibility of vascular lesion (Fig. 2). Angiography was performed for checking up the possibility of pseudoaneurysm or active bleeding. However, pelvic angiography only detected a hypervascular tumor with blood supply originating from right external iliac artery and inferior epigastric artery (Fig. 3). Biopsy was suggested but patient refused for high risk of bleeding. Thus, we performed surgical tumor resection with negative surgical margin. During the operation, a 15 cm × 12 cm × 5 cm tumor was resected, which was adherent to right spermatic cord without invasion.

**Fig. 4:**
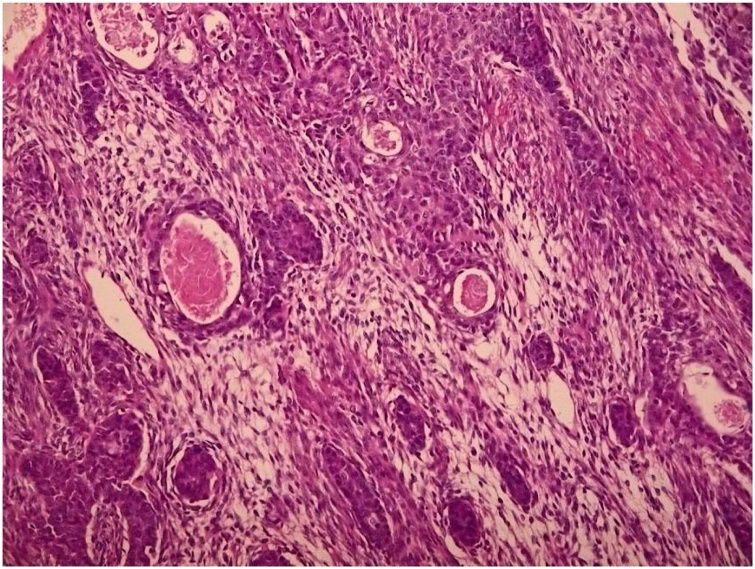
Microscopic appearance of synovial sarcoma. The tumor consisted of spindle-to-avoid cells and tubular glandular structures in addition to hemorrhage and necrosis. (hematoxylin-eosin, original magnification: 200×).

Microscopically, the tumor was composed of spindle-to-ovoid tumor cells with areas of tubular glandular structures. More rounded cells and increased mitotic activity were also observed in poorly differentiated areas (Fig. 4). Immunohistochemically, the tumor cells were positive for transducin-like enhancer protein 1 and cytokeratin and focally positive for BCOR. The tumor cells were negative for S100 and SOX10. Molecular analysis conducted through fluorescence in situ hybridization (FISH) showed SS18 gene rearrangement. According to these findings, a primary biphasic SS was diagnosed. He recovered well in the postoperative course without major complications.

The patient refused adjuvant radiation or chemotherapy owing to personal reasons. He had been followed up in clinic for 12 months, and no recurrence was observed.

## 3 Discussion

SS constitutes 5%–10% of STS. Despite its name, SS can originate from different organs, such as large joints, heart, lung, and abdominal wall. The most primary sites are extremities (68.7%) and trunk (15.7%) [4]. The cellular origin of SS is disordered mesenchymal stem cells that have undergone self-renewal dysregulation mediated by the SS18-SSX fusion protein [5]. SS has been reported to frequently affect adolescents and young adults with a median age of 35 years, with slight male predominance [4]. The 5-year survival rate ranges from 36% to 76%. A study demonstrated that female sex, nonblack race, extremity tumor, localized tumor, and tumor size smaller than 5 cm were associated with longer survival. The most metastatic sites are lung and bone [6].

Treatment options for SS include surgery, chemotherapy, radiotherapy, targeted therapy, and novel therapies. Wide-margin surgical resection remains the essential treatment for localized tumors. However, no consensus exists regarding the systemic treatment for SS. Recent studies have adequately discussed insight into neoadjuvant chemotherapy. Le Cesne et al. pooled two phase III clinical trials and revealed that adjuvant chemotherapy provided no overall survival or relapse-free survival benefits in completely resected SS [7]. A phase II trial focused on perioperative ifosfamide and doxorubicin for high-grade STS, which included 20 patients with SS, reported that the 5-year overall survival rate was 82.6% [8]. Pazopanib, a multitargeted tyrosine kinase inhibitor, was approved for STS treatment. In the PALETTE trial, pazopanib showed progression-free survival benefit in patient with metastatic STS, including those with SS [9]. Novel therapies targeting molecular and genetic pathways of SS are under research. A phase I trial of an SYT-SSX-derived peptide vaccine in six patients with SS was conducted in 2005 [10]; the trial demonstrated that the vaccine was well tolerated by all patients and it stabilized the disease in one patient. Recent studies have reported that retinoic acid and its derivatives inhibited growth signals and induced differentiation of SS cell lines both in vitro and in vivo, which could be a potential molecular target for treating SS [11]. A phase II trial of an epidermal growth factor receptor inhibitor was conducted on patients with SS who exhibited EGFR overexpression. Adjuvant radiation therapy can be administered through various approaches, including external beam therapy, brachytherapy, and intensity-modulated radiation therapy. Radiation therapies increased local control rates in patients with high-grade SS irrespective of ways of radiation therapy [12].

Four studies reporting inguinal SS in the literature were reviewed (Table 1). Hiede et al. [13] presented one case of SS in groin and no evidence of recurrence after surgical resection. Naito et al. [14] reported a typical biphasic-type SS from femoral neck, who had similar manifestations as our case. Open biopsy showed hematoma and angiography revealed abundant tumor blood supply from external iliac artery. No recurrence was observed after neoadjuvant chemo-radiotherapy and surgical resection. Xu et al. [3] reported a 68-year-old woman of inguinal monophasic synovial sarcoma with poor prognosis. Lung and spleen metastasis were noted 15 months after surgical resection, and she died of disease progression after 24-month follow-up. Wang et al. [15] reported a 53-year-old man with SS originating from spermatic cord. There is no evidence of recurrence after radical orchiectomy and adjuvant chemoradiotherapy for 36-month follow-up.

**Table 1:** Details of primary inguinal synovial sarcoma in literature review.

Study	Age	Sex	Management	Diagnosis	Follow up
Heide et al. 1988 [13]	47	Male	Surgical excision	SS in groin	No evidence of recurrence after 56-month follow-up
Naito et al. 2000 [14]	19	Female	Neoadjuvant chemoradiotherapy + surgical excision	Biphasic SS from femoral neck	No evidence of recurrence after 24-month of follow-up
Xu et al. 2010 [3]	68	Female	Surgical excision	Monophasic SS in groin	Lung and spleen metastases were observed 15 months after surgery, and patient died 24 months after surgery
Wang et al. 2015 [15]	53	Male	Surgical excision + adjuvant chemoradiotherapy	Monophasic SS of spermatic cord	No evidence of recurrence after 36-month of follow-up

In our case, the initial impressions of large inguinal tumor were hematoma or pseudoaneurysm. Preoperative angiography assisted in differentiating the lesion, evaluating tumor vessels, and preventing bleeding by embolization. We did not performed embolization in our patient due to high risk of nontargeted embolization to abdominal skin. Our patient recovered satisfactorily after surgical excision without major complications. We suggested adjuvant chemoradiotherapy, but the patient refused due to personal reasons. There was no evidence of recurrence for 12 months follow-up.

## 4 Conclusion

In conclusion, this is the first case report of biphasic SS in inguinal region. Various manifestations and rarity of SS lead us have the first impression of vascular lesion. Our study demonstrated diagnostic image, surgical views, and treatment options for SS. Complete surgical resection is main treatment. Additionally, we reviewed and summarized reported cases of inguinal SS. Additional case reports and studies are warranted to achieve a clearer understanding of diagnoses and treatments of SS in groin.

## Declaration of Competing Interest

The authors report no declarations of interest.

## Funding

This research received no specific grant from any funding agency in the public, commercial, or not-for-profit sectors.

## Ethical approval

The research related to human use has been complied with all the relevant national regulations, institutional policies, and has been approved by the Research Ethical Committee of Taipei Medical University Hospital (Case number: N201910051).

## Consent

Written informed consent was obtained from the patient for publication of this case report and accompanying images.

## Author contribution

Data collection: Wei-Chieh Chen and Po-Chien Wu.

Writing the paper: Po-Chien Wu and Ting-En Tai.

Editing the paper: Wei-Chieh Chen and Chun-Yu Lin.

## Registration of research studies

1. Name of the registry: Research Ethical Committee of Taipei Medical University Hospital.
2. Unique identifying number or registration ID: N201910051.

## Guarantor

Ting-En Tai.

## Provenance and peer review

Not commissioned, externally peer-reviewed.
